# Antimicrobial, Antioxidant, and Cytotoxic Activities of *Juglans regia* L. Pellicle Extract

**DOI:** 10.3390/antibiotics10020159

**Published:** 2021-02-04

**Authors:** Floriana D’Angeli, Giuseppe Antonio Malfa, Adriana Garozzo, Giovanni Li Volti, Carlo Genovese, Aldo Stivala, Daria Nicolosi, Francesco Attanasio, Francesco Bellia, Simone Ronsisvalle, Rosaria Acquaviva

**Affiliations:** 1 Department of Biomedical and Biotechnological Sciences, Section of Biochemistry, University of Catania, 95123 Catania, Italy; fdangeli@unict.it (F.D.); livolti@unict.it (G.L.V.); 2 Department of Human Sciences and Quality of Life Promotion, San Raffaele Roma Open University, 00166 Rome, Italy; 3 Department of Drug and Health Sciences, Section of Biochemistry, University of Catania, 95125 Catania, Italy; g.malfa@unict.it (G.A.M.); racquavi@unict.it (R.A.); 4 Department of Biomedical and Biotechnological Sciences, Section of Microbiology, University of Catania, 95123 Catania, Italy; agar@unict.it (A.G.); astivala@unict.it (A.S.); dnicolosi@unict.it (D.N.); 5 Nacture S.r.l, Spin-off University of Catania, 95123 Catania, Italy; s.ronsisvalle@unict.it; 6 Institute of Crystallography, National Research Council (CNR), 95126 Catania, Italy; f.attanasio@unict.it (F.A.); francesco.bellia@cnr.it (F.B.); 7 Department of Drug and Health Sciences, Section of Medicinal Chemistry, University of Catania, 95125 Catania, Italy

**Keywords:** antiviral, antibacterial, antifungal, DPPH, SOD-like, antitumor, polyphenols

## Abstract

The difficulty to treat resistant strains-related hospital-acquired infections (HAIs) promoted the study of phytoextracts, known sources of bioactive molecules. Accordingly, in the present study, the pharmacological activities of *Juglans regia* (L.) pellicle extract (WPE) were investigated. The antiviral effect was tested against *Herpes simplex* virus type 1 and 2, *Poliovirus* 1, *Adenovirus* 2, *Echovirus* 9, *Coxsackievirus* B1 through the plaque reduction assay. The antibacterial and antifungal activities were evaluated against medically important strains, by the microdilution method. DPPH and superoxide dismutase (SOD)s-like activity assays were used to determine the antioxidant effect. Besides, the extract was screened for cytotoxicity on Caco-2, MCF-7, and HFF1 cell lines by the 3-[4,5-dimethylthiazol-2-yl]-2,5 diphenyl tetrazolium bromide (MTT) assay. The total phenolic and flavonoid contents were also evaluated. Interestingly, WPE inhibited *Herpes simplex* viruses (HSVs) replication, bacterial and fungal growth. WPE showed free radical scavenging capacity and inhibited superoxide anion formation in a dose-dependent manner. These effects could be attributed to the high content of phenols and flavonoids, which were 0.377 ± 0.01 mg GE/g and 0.292 ± 0.08 mg CE/g, respectively. Moreover, WPE was able to reduce Caco-2 cell viability, at both 48 h and 72 h. The promising results encourage further studies aimed to better elucidate the role of WPE in the prevention of human infectious diseases.

## 1. Introduction

The emerging phenomenon of antimicrobial resistance (AMR) strongly limits the available therapeutic choices for the treatment of a large variety of infectious diseases, leading to an increased risk for severe infections, complications, and mortality [1]. Besides, AMR-related drug ineffectiveness can also significantly affect the success of major surgery and cancer chemotherapy [2,3]. Indeed, the precarious immune system of these patients promotes the development of opportunistic infections, which, in the absence of appropriate therapies, could result fatally. Thus, the concomitant presence of vulnerable individuals and the constant inclination to harboring pathogens make the hospital a high-risk environment for infectious diseases [4]. In this regard, the etiological agents responsible for hospital-acquired infections (HAIs) include bacteria, fungi, and viruses [5]. A recent surveillance study showed that most HAIs are provoked by *Escherichia coli* (18%), *Staphylococcus aureus* (12%), and *Klebsiella* spp. (9%), followed by *Pseudomonas aeruginosa* (8%), *Enterococcus faecalis* (8%), coagulase-negative staphylococci (7%), *Enterobacter* spp. (5%), *Enterococcus faecium* (4%), *Proteus* spp. (3.2%), and other *Enterococcus* spp. (3%). Moreover, this study revealed that a certain percentage of HAIs (6.3%) is due to fungal colonization and invasion, mainly to *Candida albicans* which account for 3.2% of the cases [6]. However, the concrete possibility to isolate antimicrobial-resistant strains among these pathogens could seriously compromise the efficacy of the treatment, with a consequent worsening of clinical conditions and, in extreme cases, even leading to a fatal outcome.

Regarding viruses, viral opportunistic infections occur when the host defenses are reduced, a condition frequently observed in hospitalized patients [7]. In this respect, the most common viruses involved in HAIs include *Herpes simplex* viruses 1 and 2 (HSV-1 and HSV-2), enteroviruses such as coxsackieviruses A and B, echoviruses, polioviruses, and adenoviruses [7,8,9]. 

In this context, much attention has been paid to the pharmacological role of natural extracts, as a source of bioactive compounds, able to hinder both pathogen and cancer cell physiology [8]. Concerning that, it has been shown that the crude extracts of the different tissues of walnut (kernel, shell, husk, bark, root, leaves, and septum) are endowed with outstanding pharmacological activities, including the anti-inflammatory, blood clotting, neuroprotective, antioxidant, antiproliferative, and antimicrobial properties [9,10,11,12,13,14,15]. 

In recent work, our group demonstrated a double effect of walnut septum extract, which was able to counteract both human glioblastoma cell survival and bacterial growth [16]. On the other hand, the biological effects of the edible portion covering the walnut kernel (pellicle) remain poorly investigated. In our previous paper, we characterized the chemical constituents of walnut pellicle extract (WPE), through ultra-performance liquid chromatography–tandem mass spectrometry (UPLC-Ms/Ms) method. It is worth noting that the phytochemical profile of an extract can vary depending on the geographical area in which the relative plant has grown. Accordingly, *J. regia* chemical composition is strictly related to cultivar and climate area [17]. Indeed, our chemical analysis showed a high content of active compounds, mostly phenols, and hydrolyzable tannins. Interestingly, we also demonstrated the ability of WPE to inhibit the growth and biofilm formation of coagulase-negative staphylococci (CoNS). Furthermore, this extract proved to be effective in eradicating the biofilm previously formed by CoNS [18]. The promising results obtained led us to deepen the antimicrobial properties of this extract, by studying its effect on the most common etiological agents involved in HIAs. 

Besides, since opportunistic infections can significantly affect the clinical outcome of oncologic patients, it could be useful to find novel antimicrobial agents also able to prevent cancer cell proliferation. Accordingly, the cytotoxic effect of WPE was evaluated on two tumoral (human colorectal adenocarcinoma cell line Caco-2 and human breast cancer cell line MCF7) and one primary (human foreskin fibroblast cell line HFF1) cell lines. Finally, by DPPH and the Folin–Ciocalteau assays, we analyzed the antioxidant properties and the total phenol content of WPE, respectively. 

## 2. Results

### 2.1. Cytotoxicity on HEp-2 and Vero Cell Lines

The 50% cytotoxic doses (CD_50_) of WPE on HEp-2 and VERO cells were 25.00 and 28.00 µg/mL, respectively (Table 1). The non-cytotoxic doses were used to perform the antiviral evaluation.

### 2.2. Antiviral Activity

Results obtained from our screening demonstrated that WPE had an inhibitory effect on HSV-1 and HSV-2 replication at doses below the CD_50_. The ID_50_ values were found to be 10 and 8 µg/mL for HSV-1 and HSV-2, respectively. The antiviral effect of WPE was compared to acyclovir, to which HSV-1 and HSV-2 showed an ID_50_ of 0.12 and 0.36 µg/mL, respectively. WPE was ineffective against *Echovirus* 9 (ECHO 9), *Poliovirus* 1 (Polio 1), *Coxsackievirus* B1 (Coxsackie B1), and *Adenovirus* 2 (Adeno 2) (Table 1). The study of the effect of the compound on neutralization of viral infectivity demonstrated that WPE had not virucidal activity against all the viruses tested.

### 2.3. Antibacterial Activity

Based on minimal inhibitory concentration (MIC) values, which ranged from 8.59 to 275.00 µg/mL, the Gram-positive strains were more sensitive to WPE than the Gram-negative ones. It is worth to note that WPE, at the concentration of 275.00 µg/mL, was able to inhibit the growth of five clinical strains resistant to ciprofloxacin (*E. faecium* 018/040, *E. coli* 024/040, *E. coli* 025/040, *Klebsiella pneumoniae* (*K. pneumoniae*) 035/040 and *P. aeruginosa* 028/040). The results of the antibacterial activity are shown in Table 2.

### 2.4. Antifungal Activity

The results of the antifungal activity of WPE, compared to fluconazole, are summarized in Table 3. WPE showed good inhibitory activity against most of *C. albicans* strains, with MIC values ranging from 17.18 to 275.00 µg/mL. The MICs for non-*albicans Candida* strains ranged from 137.50 to 275.00 µg/mL. *Candida krusei* (*C. krusei*) 002/020, *C. krusei* ATCC 6258, *Candida glabrata* (*C. glabrata*) ATCC 2001, and *Candida tropicalis* (*C. tropicalis*) 011/020 were resistant to WPE. All the tested strains, except three non-*albicans Candida* species, were susceptible to the standard antifungal agent fluconazole. 

For WPE, the minimal fungicidal concentration (MFC)/MIC ratio was not calculated, since all the MFC values and some MIC values were higher with respect to the concentration range used. Regarding fluconazole, MFC/MIC ratio was obtained for *C. albicans* 007/040 and *C. albicans* ATCC 90028, *C. glabrata* 005/020, and *Candida parapsilosis* (*C. parapsilosis*) ATCC 90018 with a fungistatic effect (MFC/MIC ratio >4.00).

### 2.5. Antioxidant Activity and Total Phenolic and Flavonoid Contents

The extract inhibited superoxide anion formation in a dose-dependent manner. The scavenger effect showed an IC_50_ value of 80 ± 0.51 µg/mL, comparable with the IC_50_ value of 50 mU ± 0.85 of the positive control superoxide dismutase (SOD). The antioxidant activity, analyzed with a DPPH test, showed a concentration-dependent free radical scavenging capacity, with an IC_50_ value of 48.35 ± 1.7 µg/mL, equivalent to 15 mM ± 0.62 of Trolox. The total phenol and flavonoid content assays showed values of 0.377 ± 0.01 mg gallic acid equivalent (GE)/g extract and 0.292 ± 0.08 mg catechin equivalent (CE)/g extract (Table 4). These results are consistent with our previous chemical analysis which revealed the presence of this class of compounds [18]. The main UPLC-Ms/Ms-identified molecules and their relative pharmacological activities are reported in Table 5. 

### 2.6. Cytotoxicity on CaCo-2, MCF-7, and HFF1 Cell Lines

**Figure 1:**
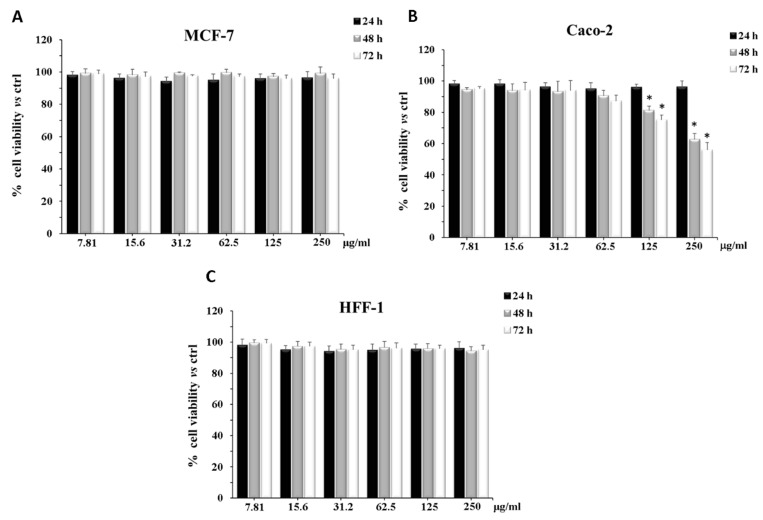
Cell viability of MCF-7 (**A**), Caco-2 (**B**), and HFF-1 (**C**) cells, untreated and treated for 24 h, 48 h, and 72 h with ethanolic extract of *Juglans regia* at different concentrations. Values are the mean ± SD of four experiments in triplicate. Statistically significant differences are indicated: * *p* < 0.05 versus control at the same incubation time.

WPE did not modify the cell viability of MCF-7 and HFF1 cells independently of the treatment time and the concentration used (Figure 1A,C). Conversely, the treatment of Caco-2 cells with different concentrations of the extract reduced cell viability after 48 h and 72 h of exposure, in a dose-dependent manner and the inhibitory effects, at 250 µg/mL, reached a value of about 40% and 50%, respectively (Figure 1B). 

## 3. Discussion

Hospital-acquired infections represent a serious complication, often arising during the convalescence period in a medical facility [43]. It has been reported that HAIs amount to the first cause of death among hospitalized patients. To prevent these injurious events, the Centers for Disease Control and Prevention (CDC) constantly monitor the clinical practices correlated to an increased risk for microorganism contamination and invasion, including ventilator-associated pneumonia, catheter-associated urinary tract infections, bloodstream infections, and surgical site infections [44]. These illnesses can be caused by different pathogens, belonging to bacteria, fungi, or virus’s family. Accordingly, infectious diseases can significantly compromise the clinical outcome of the patients, including the oncologic ones. The increasing development of resistant mechanisms to the most commonly used drug promoted the research of new bioactive chemical compounds from phytoextracts. Concerning that literature data demonstrated the antimicrobial and the antiproliferative effects of walnut extracts [11,16,45,46,47]. However, despite the great attention paid to *J. regia* extracts, the knowledge on the biological effects of the edible pellicle is still limited. 

In our previous work, we proved an antibacterial and antibiofilm activity of the present extract against CoNS. Furthermore, by UPLC-Ms/Ms, we chemically analyzed the phytoextract, revealing the presence of several biologically active molecules (Table 5) [18]. These results led us to hypothesize further antimicrobial properties. Indeed, in the present study, we found that WPE exerts an interesting activity against HSV-1 and HSV-2, with a concentration-dependent antiviral effect. It was demonstrated that natural extract rich in polyphenols are capable to directly block virus attachment to host-cell [48,49] or to interfere with the early phases of the replicative cycle [50]. Accordingly, the anti-herpes effect may be attributed to a synergistic effect of different compounds, belonging to phenols, flavonoids, and sterols. For example, among flavonoids, rutin and quercetin exerted potent anti-herpetic activity, both showing an EC_50_ of 5 μM against HSV-1, although only quercetin inhibited HSV-2 infection, with an EC_50_ of 35 μM [31,32]. A number of reports highlighted the antiviral role of quercetin against HSV. Hung et al. demonstrated the ability of quercetin to inhibit HSV-1 infection of Vero cells [42]. A further study showed that this molecule is able to modulate the expression of HSV proteins, such as the viral glycoprotein D (gD), an essential protein for the attachment of virus on host cell membrane during the infectious process, and ICP0, a viral protein expressed in the early phases of the HSV-1 replication cycle. Furthermore, the authors also demonstrated that quercetin acts at the genic level, downregulating the correlated gene-replication such as ICP0, UL13, and UL52 [51]. The WPE contains other compounds [18], including protocatechuic acid, myricetin [19], gallic acid, ellagic acid, kaempferol [22], and β-sitosterol [36], which were also associated with the anti-HSV activity. Interestingly, an *in-silico* analysis showed that tris-juglone, one of the previously determined constituents of WPE (Table 5) [18], showed antiviral activity against the SARS-CoV-2 virus by inhibiting cathepsin L, a lysosomal cysteine endopeptidase involved in the activation of heparinase [39]. The indirect block of this enzyme reduces the egress of the virus from the host cell. Since cathepsin L plays the same role in HSV-2, we can hypothesize that the HSV-inhibitory effect of WPE could be similarly mediated by tris-juglone [52]. 

Furthermore, WPE was more efficient in reducing bacterial growth of Gram-positive strains compared to those Gram-negative, as indicated by the lower MIC values. This dissimilar effect reflects differences in the cell wall composition between the two bacterial groups [16,53]. Indeed, the Gram-negative bacteria are characterized by a highly selective external membrane to the passage of the molecules, which makes these bacteria more resistant to drugs [53,54,55]. Nevertheless, as reported by Saraiva et al., since several bacteria strains showed MIC values under the concentration of 500 µg/mL, WPE can be considered an active antimicrobial agent [56]. 

*Candida* species are also responsible for healthcare-related infections both in immunocompetent and immunocompromised hosts [57,58]. Although new synthetic drugs are generally active against resistant *Candida* species, their administration can cause toxicity, interactions with other drugs, and inadequate bioavailability [59,60,61]. Experimental evidence supported the anticandidal activity of different extracts from *J. regia*, including leaf, bark, and root [62,63,64,65]. In agreement with these data, our findings showed an antifungal activity of WPE against different *Candida* species, with MICs ranging from 17.18 to 275.00 µg/mL. This effect could be attributed to the presence of the flavonoids quercetin, myricetin, and rutin [34]. Besides, in our previous chromatographic analysis, we identified the flavonol avicularin. This compound was isolated from *Juglans sinensis* leaves [66]. Da Silva Sa et al. demonstrated that this compound is able to inhibit *C. albicans* growth in concentrations of 2 to 16 µg/mL [37]. Concerning non-*albicans Candida* species, the extract was less efficient in reducing the growth of these pathogens. This effect could be due to both a reduced permeability of these yeasts to natural extracts and to their higher capacity to produce biofilm [67,68]. 

Taken together, these results clearly showed good antimicrobial effects of WPE, revealing its efficacy against different pathogens. In the oncologic field, the development of opportunistic infections is considered a common side effect of chemotherapy [69]. According to this concept, we evaluated the potential antiproliferative action of the extract, by treating two different cancer cell lines (MCF-7 and Caco-2 cells) and a primary fibroblast cell line (HFF-1) with increasing concentrations of WPE, for 24 h, 48 h, and 72 h. Interestingly, after a prolonged period (48 h and 72 h) the treatment with WPE significantly reduced Caco-2 cell viability in a dose-dependent manner. Considering that the pellicle of walnut is edible, being concomitantly ingested with the fruit and that the gastrointestinal tract is the most exposed district to the action of dietary ingredients, it is possible to hypothesize a benefic effect of this portion of the walnut, rich in polyphenols, at the intestinal level [70]. In this respect, literature data showed the ability of a large variety of classes of dietary polyphenols, including rutin, quercetin, myricetin, ellagic acid [30], and avicularin [38], all present in our extract, to affect glucose transport in Caco-2 cells via both facilitated transport proteins (GLUT 1 and 2) and sodium-dependent glucose transporter 1 (SGLT1). Several natural extracts, usually rich in these molecules, were also found to inhibit glucose uptake in Caco-2 cells through these mechanisms [38,71]. Being the main cellular energetic source, glucose plays a key role also in cancer cell growth [72]. It has been demonstrated that the inhibition of glucose uptake is able to arrest cancer progression [73]. Therefore, in our case, the cytotoxic effect of WPE on Caco-2 cells could be attributed to the reduced glucose internalization, mediated by the synergistic action of polyphenolic compounds. Conversely, the inefficacy of WPE on MCF-7 cells confirmed the results obtained in our previous studies, which showed a greater drug resistance of these cells, induced by the breast cancer resistance protein (BCRP) expression [74]. Notably, WPE did not determine any cytotoxic effect on the primary HFF-1 cells. 

The protective role of WPE was further explored by evaluating its potential scavenger activity against reactive oxygen species (ROS). As expected, the extract inhibited superoxide anion formation similarly to the SOD enzyme, also showing the ability to bleach the stable DPPH radical. The free radical-scavenging activity is strictly related to the high content of phenols and flavonoids, as determined by the Folin–Ciocalteu method (Table 4). These results are consistent with other studies focusing on *J. regia* pellicle extracts, which revealed a high amount of phenols and flavonoids, mostly in yellow pellicle than in red ones [75,76]. 

## 4. Materials and Methods 

### 4.1. Chemical Reagents

All chemicals were purchased from Sigma-Aldrich S.r.l. (Milano, Italy), except those mentioned elsewhere.

### 4.2. Plant Material and Preparation of the Extract

**Figure 2:**
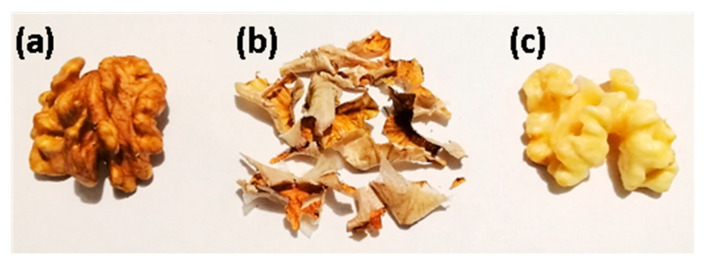
(**a**) Kernel; (**b**) pellicle; (**c**) kernel without the pellicle.

Walnuts were collected on September 2018 in Modica (Ragusa, Italy; Latitude 36°51′49.71″ N, Longitude 14°45′56.00″ E, Altitude 390 m) (Figure 2). The specimen was authenticated by the botanist and co-author Rosaria Acquaviva. A voucher specimen was deposited in the herbarium of the Department of Drug and Health Sciences, University of Catania. The extract was obtained by maceration of 10 g of walnuts pellicle in 50 mL of analytical reagent grade ethanol (ARGE) for 48 h, under constant shaking, at room temperature. The process was repeated four times. The four aliquots were reunited, filtered, and evaporated to dryness at 40 °C with a rotatory evaporator (Stuart RE300) 0.275 g of dry extract were initially dissolved in 10 mL of ARGE and further diluted in the medium before use to achieve the final concentration needed. The dilution of walnut pellicle extract (WPE) contained a maximum concentration of 0.01% (*v*/*v*) ethanol.

### 4.3. Cytotoxicity Assay on HEp-2 and Vero Cell Lines

The cytotoxicity of WPE was determined by measuring the effect on cell morphology after 24, 48, and 72 h, by light microscopy and cell viability evaluation through the 3-[4,5-dimethylthiazol-2-yl]-2,5 diphenyl tetrazolium bromide (MTT) assay in 96-well tissue culture microplates (Corning). The CD_50_ was defined as the concentration of the extract that inhibited 50% cell growth when compared with the control cultures [77].

### 4.4. Antiviral Activity

HSV-1 (ATCC^®^ VR-260^TM^) and HSV-2 (ATCC^®^ VR-734^TM^) were propagated in VERO cells (ATCC^®^ CCL-81™); ECHO 9 (ATCC^®^ VR-39™) was propagated in LLC-MK2 cells (ATCC^®^ CCL-7™); Polio 1 (ATCC^®^ VR-58^TM^), Coxsackie B1 (ATCC^®^ VR-687™) and Adeno 2 (ATCC^®^ VR-1079AS/RB™) were propagated in HEp-2 cells (ATCC^®^ CCL-23™). Cells were kept in a humidified 5% carbon dioxide atmosphere at 37 °C and grown in Dulbecco’s Modified Eagle Medium (D-MEM) supplemented with 10% heat-inactivated fetal calf serum (FCS), 200 µg/mL of streptomycin, and 200 units/mL of penicillin G (Gibco^TM^). For the virus tested, working stock solution was prepared as cellular lysates using D-MEM with 2% heat-inactivated FCS, 0.2 g L^−1^ of streptomycin, and 200 U mL^−1^ of penicillin G (maintenance medium). The antiviral activity of WPE was evaluated by 50% plaque reduction assay, as previously described [77]. The concentration of extract required to inhibit 50% virus plaque formation was expressed as the 50% inhibitory dose (ID_50_). It was calculated by dose–response curves and linear regression. Mock-infected and infected cells without compound served as cell and virus control, respectively. To test possible virucidal activity, 0.5 mL of viral suspension (10^6^ PFU/mL) and MEM containing the extract (10 x the ID_50_) were mixed and incubated for 1 h at 37 °C. Infectivity was determined by plaque assay after dilution of the virus below the inhibitory concentration. The effect of WPE was compared to the antiviral agent acyclovir.

### 4.5. Antibacterial Activity

Antibacterial activity of WPE was tested against thirty-two clinical isolates from different sources and eight standard strains, purchased from the American Type Culture Collection (Rockville, MD, USA). The antibacterial activity of WPE was determined by the broth microdilution method in sterile 96-well microplates (Corning). All the procedures were carried out with reference to the standard procedures of the Clinical and Laboratory Standards Institute (CLSI) [78]. The dry extract was solubilized in ethanol and diluted in the 1:100 ratio in cation adjusted Mueller-Hinton broth (CAMHB) (Becton Dickinson). After filtration with a 0.22 μm filter (Millipore), the stock solution was subjected to serial twofold dilutions in concentrations ranging from 0.53 to 275.00 μg/mL. Isolated colonies grown on Mueller Hinton agar plates (Oxoid) were suspended in 0.85% NaCl, to obtain turbidity equivalent to 0.5 McFarland Standard (1.5 × 10^8^ CFU/mL). The turbidity of the suspensions was determined through a spectrophotometer at λ = 600 nm (Synergy HT—Biotech). The final bacterial concentration per well was 5 × 10^5^ CFU/mL. Ciprofloxacin was used as a positive control. Ten percent ethanol and plant extract without bacterial suspension was used as the negative controls. The Minimal Inhibitory Concentration (MIC) was defined as the lowest concentration at which there was no visible growth after incubation at 37 °C for 18–24 h. Results are expressed as the mean of four experiments.

### 4.6. Antifungal Activity

The antifungal activity of WPE extract was determined against seven *C. albicans* and four non-*albicans Candida* clinical strains, isolated from vaginal swabs. The swabs were anonymous and no information about patients was reported to the laboratory. *C. albicans* ATCC 90028, *C. krusei* ATCC 6258, *C. glabrata* ATCC 2001, *C. tropicalis* ATCC 750, and *C. parapsilosis* ATCC 90018 were purchased from the American Type Culture Collection (Rockville, MD, USA) and used as control strains. The assay was performed according to the standard procedures of CLSI M27-A3 [79]. The culture medium used for the experiments was bicarbonate-free RPMI 1640 with L-glutamine, buffered to pH 7.0 with 0.165 M morpholinepropanesulfonic acid. *Candida albicans* strains were cultured on Sabouraud dextrose agar (Oxoid, Thermo Fisher Scientific, Waltham, MA, USA) and incubated for 48 h at 35 °C. The 0.5 McFarland suspensions were prepared in sterile saline solutions and the turbidity was evaluated through a spectrophotometer at λ = 530 nm. This procedure yielded a concentration between 1 × 10^6^ and 5 × 10^6^ cells/mL. Subsequently, the suspensions were diluted in the 1:100 ratio in RPMI 1640 medium (RPMI). Fluconazole was used as the positive control and obtained in concentrations ranging from 0.031 to 64.00 μg/mL. WPE was dissolved in ethanol and diluted in the 1:100 ratio in RPMI. Two-fold serial dilutions were performed to obtain a concentration series ranging from 0.53 to 275.00 μg/mL. One hundred microliters of standardized fungal suspensions were deposited in each well, resulting in a final concentration from 5 × 10^2^ to 2.5 × 10^3^ cells/mL per well. The MICs were read by a spectrophotometer at λ = 405 nm and defined as the lowest concentrations resulting in a 50% reduction in growth compared with that of the drug-free growth control, after incubation at 35 °C for 48 h. Besides, minimum fungicidal concentration (MFC) and MFC/MIC ratio were determined. This allowed us to verify if the natural extract had a fungistatic or fungicidal effect. Results are expressed as the mean of four experiments.

### 4.7. Antioxidant Activity and Determination of Total Phenolic and Flavonoid Contents

Radical scavenging activity of *J. regia* pellicle extract against stable DPPH• (2,2-diphenyl-2-picrylhydrazyl hydrate) was determined spectrophotometrically by the modified method described in Tenuta and collaborators and compared to Trolox (30 µM) as reference compound [80]. The scavenger effect of the pellicle extract on superoxide anion (SOD-like activity) was performed as previously reported [81]. Results are expressed as the percentage of inhibition of NADH oxidation, and SOD (80 mU) was used as a reference compound. The results for both determinations were obtained from the average of three independent experiments, and were reported as the mean 50% inhibitory concentration (IC_50_) ± S.D. Using the Folin–Ciocalteau total phenols procedure, as described by Malfa et al. 2020, the total amount of total phenolic compounds was determined spectrophotometrically. The polyphenols concentration detected was estimated as gallic acid equivalent (GE) and expressed in mg GE/g extract. The flavonoid content was measured using a colorimetric assay [81] and estimated as catechin equivalent (CE) and expressed in mg CE/g extract. Each result represents the mean ± S.D. of three experimental determinations.

### 4.8. Cell Culture 

Human colorectal adenocarcinoma cells (Caco-2), breast cancer cells (MCF-7), and human foreskin fibroblast (HFF1) were obtained from the American Type Culture Collection (ATCC, Rockville, MD, USA). Caco-2 were cultured in Dulbecco’s modified Eagle’s medium supplemented with 10% fetal calf serum, 1 mmol/L sodium pyruvate, 2 mmol/L L-glutamine, streptomycin (50 mg/mL), and penicillin (50 U/mL). MCF-7 were cultured in RPMI medium containing 10% fetal bovine serum, 100 U/mL penicillin, and 100 mg/mL streptomycin. HFF1 were cultured in Dulbecco’s modified Eagle’s medium supplemented with 15% fetal bovine serum, 4.5 g/L glucose, 100 U/mL penicillin, and 100 mg/mL streptomycin. HFF1 were used as in vitro human model for preliminary toxicity screening. To obtain the same experimental conditions and precision in the measurements, the cells were plated at a constant density (3 × 10^5^ cells/mL).

### 4.9. Cytotoxicity Assay on CaCo-2, MCF-7, and HFF1 Cell Lines

After 24 h of incubation in humidified atmosphere of 5% CO_2_ at 37 °C to allow cell attachment on a 96 multiwell plate (8 × 10^3^ cells/well), at sub-confluent conditions, CaCo-2, MCF-7, and HFF1 cells were treated with different concentrations of *J. regia* extract for 24 h, 48 h, and 72 h. The extract was dissolved in medium to obtain final concentrations ranging from 0.00780 to 0.25 mg/mL. This assay measures the conversion of tetrazolium salt to yield colored formazan in the presence of metabolic activity. The amount of formazan is proportional to the number of living cells. The optical density was measured with a microplate spectrophotometer reader (Titertek Multiskan, Flow Laboratories, Helsinki, Finland) at λ = 570 nm. Results are expressed as percentage cell viability with respect to control (untreated cells) [74].

### 4.10. Statistical Analysis

The IC_50_ value represents the concentration at which a substance exerts half of its maximal inhibitory effect. It is obtained through linear regression analysis and it is expressed as the mean ± standard deviation (S.D.). The linear regression analysis was performed using Origin 6.0 software.

Data are expressed as mean ± S.D. for three replicates of almost three independent experiments (i.e., biological and technical triplicates). Statistical significance was analyzed by one-way Anova test.

## 5. Conclusions

In the present study, we found that *J. regia* pellicle extract efficiently reduced HSV replication, bacterial and fungal growth, and colorectal cancer cell viability. Besides, WEP possessed interesting antioxidant properties. Our findings indicate that this extract represents a rich source of bioactive compounds, able to affect pathogen and cancer cell survival. In light of the above considerations, we can conclude that this phytoextract is endowed with several health-promoting properties. Accordingly, WPE might be useful in the prevention and control of several human diseases; however, further investigations are required to elucidate the mechanisms of action underlying biological effects and the possible application in the pharmaceutical field.

## Figures and Tables

**Table 1:** Antiviral activity of walnut pellicle extract.

	CD_50_^a^		ID_50_^b^					
	HEp-2	VERO	HSV-1	HSV-2	ECHO 9	Polio 1	Coxsackie B1	Adeno 2
**WPE (µg/mL) ^c^**	25.00	28.00	10.00	8.00	ORC ^d^	ORC	ORC	ORC
**Acyclovir (µg/mL)**	>600.00	>600.00	0.12	0.36	ORC	ORC	ORC	ORC

^a^ CD_50_, concentration which inhibited 50% cell growth compared to control cultures; ^b^ ID_50_, the concentration which inhibited 50% virus plaque formation and virus-induced cytopathogenicity. Values are mean ± S.D. (maximal S.D. estimated) for three separate assays. ^c^ WPE: walnut pellicle extract. ^d^ ORC: out of range of concentration.

**Table 2:** Antibacterial activity of walnut pellicle extract against Gram-positive and Gram-negative strains.

Bacterial Strains ^b^	Source	MIC ^a^ (µg/mL)		I.C. ^e^
		WPE ^c^	Cip ^d^	
		Range [0.53–275.00]	[0.06–32.00]	
**Gram-positive**				
*E. faecalis* 012/040	Abscess	8.59	0.50	S
*E. faecium* 019/040	Catheter cystitis	8.59	1.00	S
*S. epidermidis* 007/040	Osteomyelitis	8.59	0.031	S
*S. epidermidis* ATCC 14990	Standard	8.59	0.125	S
*E. faecalis* 015/040	Abscess	17.18	0.25	S
*S. aureus* 002/040	Endophtalmitis	17.18	0.25	S
*S. aureus* 004/040	Pneumonia	17.18	0.25	S
*E. faecalis* 013/040	Septicemia	34.37	0.50	S
*S. aureus* 005/040	Endophtalmitis	34.37	0.50	S
*E. faecalis* ATCC 29212	Standard	68.75	0.50	S
*E. faecalis* 014/040	Pneumonia	68.75	1.00	S
*E. faecium* 018/040	Catheter cystitis	275.00	8.00	R
*S. aureus* ATCC 29213	Standard	275.00	0.50	S
*S. epidermidis* 009/040	Endophtalmitis	275.00	0.015	S
*E. faecium* 017/040	Cholecystitis	>275.00	>32.00	R
*E. faecium* 020/040	Cholecystitis	>275.00	16.00	R
*E. faecium* ATCC 700221	Standard	>275.00	>32.00	R
*S. aureus* 003/040	Pneumonia	>275.00	4.00	R
*S. epidermidis* 008/040	Septicemia	>275.00	8.00	R
*S. epidermidis* 010/040	Septicemia	>275.00	8.00	R
**Gram-negative**				
*E. coli* 024/040	Cystitis	275.00	4.00	R
*E. coli* 025/040	Cystitis	275.00	8.00	R
*K. pneumoniae* 035/040	Nephritis	275.00	4.00	R
*P. aeruginosa* 028/040	Septicemia	275.00	4.00	R
*P. aeruginosa* 029/040	Pneumonia	275.00	0.125	S
*P. mirabilis* 038/040	Cystitis	275.00	0.015	S
*P. mirabilis* ATCC 7002	Standard	275.00	0.25	S
*E. coli* 022/040	Septicemia	>275.00	0.015	S
*E. coli* 023/040	Septicemia	>275.00	>32.00	R
*E. coli* ATCC 35218	Standard	>275.00	0.015	S
*K. pneumoniae* 032/040	Nephritis	>275.00	4.00	R
*K. pneumoniae* 033/040	Pneumonia	>275.00	32.00	R
*K. pneumoniae* 034/040	Pneumonia	>275.00	8.00	R
*K. pneumoniae* ATCC 700630	Standard	>275.00	0.25	S
*P. aeruginosa* 027/040	Septicemia	>275.00	0.06	S
*P. aeruginosa* 030/040	Pneumonia	>275.00	16.00	R
*P. aeruginosa* ATCC 27853	Standard	>275.00	0.25	S
*P. mirabilis* 037/040	Cystitis	>275.00	1.00	S
*P. mirabilis* 039/040	Cystitis	>275.00	0.015	S
*P. mirabilis* 040/040	Cystitis	>275.00	8.00	R

^a^ MIC: Minimal Inhibitory Concentration; ^b^ Strain numbers refer to an internal directory for bacteria. ^c^ WPE: walnut pellicle extract; ^d^ Cip: Ciprofloxacin; ^e^ I.C.: Interpretive criteria for Ciprofloxacin (CLSI M100-S28): ≤1 Susceptible (S); 2 Intermediate (I); ≥4 Resistant (R).

**Table 3:** Antifungal activity of walnut pellicle extract.

Fungal Strains ^c^	Source	WPE ^a^ (µg/mL)			FLU ^b^ (µg/mL)			
		MFC ^d^	MIC ^e^	MFC/MIC ^f^	MFC	MIC	MFC/MIC	I.C. ^g^
*C. albicans* 003/040	V.S. ^h^	>275.00	17.18	N.C. ^i^	>64.00	0.12	N.C.	S
*C. albicans* 006/040	V.S.	>275.00	17.18	N.C.	>64.00	0.12	N.C.	S
*C. albicans* 002/040	V.S.	>275.00	34.37	N.C.	>64.00	0.12	N.C.	S
*C. albicans* 001/040	V.S.	>275.00	137.50	N.C.	>64.00	8.00	N.C.	S
*C. parapsilosis* ATCC 90018	V.S.	>275.00	137.50	N.C.	2.00	0.25	8	S
*C. albicans* 005/040	V.S.	>275.00	275.00	N.C.	>64.00	0.12	N.C.	S
*C. glabrata* 005/020	V.S.	>275.00	275.00	N.C.	64.00	8.00	8.00	S
*C. parapsilosis* 018/020	V.S.	>275.00	275.00	N.C.	>64.00	16.00	N.C.	S-DD
*C. tropicalis* ATCC 750	V.S.	>275.00	275.00	N.C.	>64.00	2.00	N.C.	S
*C. albicans* 004/040	V.S.	>275.00	>275.00	N.C.	>64.00	0.50	N.C.	S
*C. albicans* 007/040	V.S.	>275.00	>275.00	N.C.	32.00	2.00	16	S
*C. albicans* ATCC 90028	V.S.	>275.00	>275.00	N.C.	16.00	0.50	32	S
*C. glabrata* ATCC 2001	V.S.	>275.00	>275.00	N.C.	>64.00	>64.00	N.C.	R
*C. krusei* 002/020	V.S.	>275.00	>275.00	N.C.	>64.00	>64.00	N.C.	R
*C. krusei* ATCC 6258	V.S.	>275.00	>275.00	N.C.	>64.00	64.00	N.C.	R
*C. tropicalis* 011/020	V.S.	>275.00	>275.00	N.C.	>64.00	32.00	N.C.	S-DD

^a^ WPE: walnut pellicle extract; ^b^ FLU: fluconazole; ^c^ Strain numbers refer to an internal directory for clinical isolates; ^d^ MFC: Minimal Fungicidal Concentration; ^e^ MIC: Minimal Inhibitory Concentration; ^f^ MFC/MIC ratio >4.00: fungistatic, MFC/MIC ratio ≤4.00: fungicidal; ^g^ I.C.: Interpretive Criteria for Fluconazole (CLSI M27-A3): ≤8.00 µg/mL Susceptible (S); 16.00–32.00 µg/mL Susceptible-dose-dependent (S-DD); ≥64.00 µg/mL Resistant (R); ^h^ V.S.: vaginal swab; ^i^ N.C.: not calculated; note that MFC/MIC ratio could not be calculated for *C. albicans* and non-*albicans Candida* strains with a MFC and MIC values higher of lower with respect to the concentration range used.

**Table 4:** Antioxidant activity and total phenolic and flavonoid contents of walnut pellicle extract.

Sample	SOD-like Activity	DPPH Test	Total Phenolic	Total Flavonoids
	IC_50_ (μg/mL)		Gallic Acid (mg/g)	Catechin (mg/g)
WPE ^a^	80 ± 0.51	48.35 ± 1.7	0.377 ± 0.01	0.292 ± 0.08
SOD ^b^	50 mU ± 0.85	-	-	-
Trolox	-	15 mM ± 0.62	-	-

^a^ WPE: walnut pellicle extract; ^b^ SOD: superoxide dismutase.

**Table 5:** Chemical compounds identified from walnut pellicle extract through UPLC-Ms/Ms [18] and related pharmacological activities.

Chemical Name	Chemical Class	Chemical Structure	RT * (min)	m/z (g/mol)	Pharmacological Activities
Protocatechuic acid	Benzoic acid and Phenol derivatives	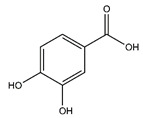	35.09	154.027	Antiviral [19] Antibacterial [20] Antioxidant [21]
Gallic acid	Benzoic acid and Phenol derivatives	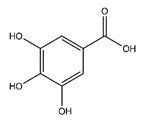	18.60	170.021	Antiviral [22] Antibacterial [23] Antioxidant [21]
Ferulic acid	Cinnamic acids and derivatives	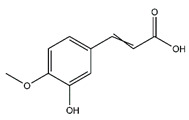	36.20	194.058	Antibacterial [23] Antioxidant [24]
Sinapate	Cinnamic acids and derivatives	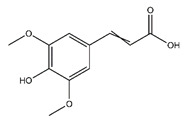	n.r.	224.210	Antibacterial, Antifungal, Antitumor [25]
Palmitic acid	Fatty acids	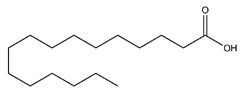	12.43	256.240	Antioxidant [26,27] Antitumor [28]
Oleic acid	Fatty acids	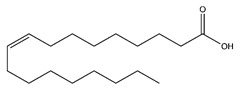	37.24	282.256	Antioxidant [26,27] Antitumor [28]
Ellagic acid	Tannins	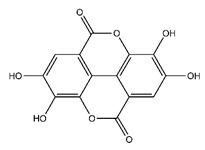	33.20	302.006	Antiviral [22] Antioxidant [29] Antitumor [30]
Quercetin	Flavonoids	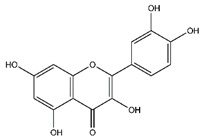	33.97	302.043	Antiviral [31,32] Antibacterial [33] Antifungal [34] Antioxidant [24] Antitumor [30]
Myricetin	Flavonoids	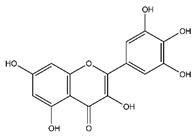	33.78	318.038	Antiviral [19] Antifungal [34] Antitumor [30]
Chlorogenic acid	Cinnamate ester derivatives	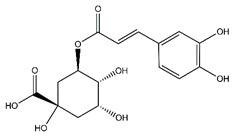	39.49	354.095	Antioxidant [35]
Beta-sitosterol	Steroids and steroid derivatives	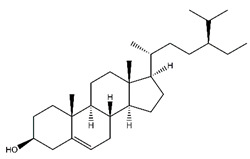	33.89	411.386	Antiviral [36]
Kaempferol-arabinoside	Flavonoids	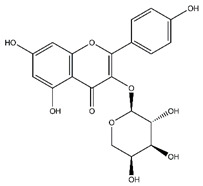	n.r.	418.080	Antiviral [22]
Tocopherol	Prenol lipids	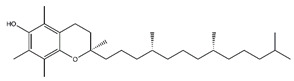	36.07	430.381	Antioxidant [26]
Avicularin	Flavonoids	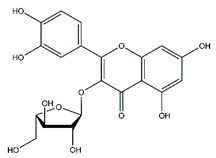	20.08	434.085	Antifungal [37] Antitumor [38]
Tris-juglone	Phenanthrenes and derivatives	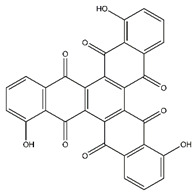	36.94	516.048	Anti-SARS-CoV-2 (*in-silico* analysis) [39]
(+)-Procyanidin B2	Flavonoids	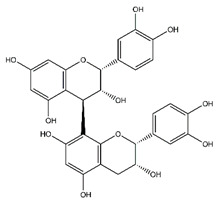	38.25	578.520	Antibacterial, Antioxidant, Antitumor [40]
Rutin	Flavonoids	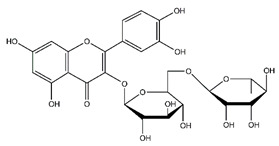	45.11	610.153	Antibacterial [41] Antiviral [31,32,42] Antifungal [34] Antioxidant [24] Antitumor [30]

Notes: * RT: retention time; n.r.: not reported. The retention time of the compounds was not reported, due to their low concentrations in the extract.

## Data Availability

The data presented in this study are available on request from the corresponding author.

## References

[1] Hay S.I., Rao P.C., Dolecek C., Day N.P.J., Stergachis A., Lopez A.D., Murray C.J.L. (2018). Measuring and mapping the global burden of antimicrobial resistance. BMC Med..

[2] Raymond D.P., Kuehnert M.J., Sawyer R.G. (2002). Preventing Antimicrobial-Resistant Bacterial Infections in Surgical Patients. Surg. Infect..

[3] Zhou S., Fan L., Wang Z., Wang Q., Xiong Z., Xu Y., Li D. (2018). Increasing rates of Acinetobacter baumannii infection and resistance in an oncology department. J. Cancer Res. Ther..

[4] Chng K.R., Li C., Bertrand D., Ng A.H.Q., Kwah J.S., Low H.M., Tong C., Natrajan M., Zhang M.H., MetaSUB Consortium (2020). Cartography of opportunistic pathogens and antibiotic resistance genes in a tertiary hospital environment. Nat. Med..

[5] Khan H.A., Baig F.K., Mehboob R. (2017). Nosocomial infections: Epidemiology, prevention, control and surveillance. Asian Pac. J. Trop. Biomed..

[6] Weiner-Lastinger L.M., Abner S., Benin A.L., Edwards J.R., Kallen A.J., Karlsson M., Magill S.S., Pollock D., See I., Soe M.M. (2019). Antimicrobial-resistant pathogens associated with pediatric healthcare-associated infections: Summary of data reported to the National Healthcare Safety Network, 2015–2017. Infect. Control. Hosp. Epidemiol..

[7] Cabrera-Cancio M.R. (2012). Infections and the Compromised Immune Status in the Chronically Critically Ill Patient: Prevention Strategies. Respir. Care.

[8] Genovese C., Acquaviva R., Ronsisvalle S., Tempera G., Malfa G.A., D’Angeli F., Ragusa S., Nicolosi D. (2020). In vitro evaluation of biological activities of Orobanche crenata Forssk. leaves extract. Nat. Prod. Res..

[9] Papoutsi Z., Kassi E., Chinou I., Halabalaki M., Skaltsounis L.A., Moutsatsou P. (2008). Walnut extract (Juglans regia L.) and its component ellagic acid exhibit anti-inflammatory activity in human aorta endothelial cells and osteoblastic activity in the cell line KS483. Br. J. Nutr..

[10] Jahanban-Esfahlan A., Ostadrahimi A., Tabibiazar M., Amarowicz R. (2019). A Comparative Review on the Extraction, Antioxidant Content and Antioxidant Potential of Different Parts of Walnut (Juglans regia L.) Fruit and Tree. Molecules.

[11] Salimi M., Ardestaniyan M.H., Kandelous H.M., Saeidnia S., Gohari A.R., Amanzadeh A., Sanati H., Sepahdar Z., Ghorbani S. (2014). Anti-proliferative and apoptotic activities of constituents of chloroform extract of Juglans regialeaves. Cell Prolif..

[12] Amirou A., Bnouham M., Legssyer A., Ziyyat A., Aziz M., Berrabah M., Mekhfi H. (2018). Effects of Juglans regia Root Bark Extract on Platelet Aggregation, Bleeding Time, and Plasmatic Coagulation: In Vitro and Ex Vivo Experiments. Evid. Based Complement. Altern. Med..

[13] Nasiry D., Khalatbary A.R., Ahmadvand H., Amiri F.B.T., Akbari E. (2017). Protective effects of methanolic extract of Juglans regia L. leaf on streptozotocin-induced diabetic peripheral neuropathy in rats. BMC Complement. Altern. Med..

[14] Bakht J., Khan S., Shafi M., Iqbal A. (2017). Fractionation of crude extracts from controlled dried and commercially available stem bark of Juglans regia and their antimicrobial effects. Pak. J. Pharm. Sci..

[15] Ahmad S., Wahid M.A., Bukhari A.Q.S. (1973). Fungistatic Action of Juglans. Antimicrob. Agents Chemother..

[16] Genovese C., Cambria M.T., D’Angeli F., Addamo A.P., Malfa G.A., Siracusa L., Pulvirenti L., Anfuso C.D., Lupo G., Salmeri M. (2020). The double effect of walnut septum extract (Juglans regia L.) counteracts A172 glioblastoma cell survival and bacterial growth. Int. J. Oncol..

[17] Wu S., Ni Z., Wang R., Zhao B., Han Y., Zheng Y., Liu F., Gong Y., Tang F., Liu Y. (2020). The effects of cultivar and climate zone on phytochemical components of walnut (Juglans regia L.). Food Energy Secur..

[18] Acquaviva R., D’Angeli F., Malfa G.A., Ronsisvalle S., Garozzo A., Stivala A., Ragusa S., Nicolosi D., Salmeri M., Genovese C. (2019). Antibacterial and anti-biofilm activities of walnut pellicle extract (Juglans regia L.) against coagulase-negative staphylococci. Nat. Prod. Res..

[19] Li W., Xu C., Hao C., Zhang Y., Wang Z., Wang S., Wang W. (2020). Inhibition of herpes simplex virus by myricetin through targeting viral gD protein and cellular EGFR/PI3K/Akt pathway. Antivir. Res..

[20] Stojković D., Živković J., Sokovic M., Glamočlija J., Ferreira I.C.F.R., Jankovic T., Maksimović Z. (2013). Antibacterial activity of Veronica montana L. extract and of protocatechuic acid incorporated in a food system. Food Chem. Toxicol..

[21] Zhang Z., Liao L., Moore J., Wu T., Wang Z. (2009). Antioxidant phenolic compounds from walnut kernels (Juglans regia L.). Food Chem..

[22] El-Toumy S.A., Salib J.Y., El-Kashak W.A., Marty C., Bedoux G., Bourgougnon N. (2018). Antiviral effect of polyphenol rich plant extracts on herpes simplex virus type 1. Food Sci. Hum. Wellness.

[23] Borges A., Ferreira C., Saavedra M.J., Simões M. (2013). Antibacterial Activity and Mode of Action of Ferulic and Gallic Acids Against Pathogenic Bacteria. Microb. Drug Resist..

[24] Ho K.-V., Roy A., Foote S., Vo P.H., Lall N., Lin C.-H. (2020). Profiling Anticancer and Antioxidant Activities of Phenolic Compounds Present in Black Walnuts (Juglans nigra) Using a High-Throughput Screening Approach. Molecules.

[25] Chen C. (2016). Sinapic Acid and Its Derivatives as Medicine in Oxidative Stress-Induced Diseases and Aging. Oxidative Med. Cell. Longev..

[26] Li L., Tsao R., Yang R., Kramer A.J.K.G., Hernandez M. (2007). Fatty Acid Profiles, Tocopherol Contents, and Antioxidant Activities of Heartnut (Juglans ailanthifolia Var. cordiformis) and Persian Walnut (Juglans regia L.). J. Agric. Food Chem..

[27] Cotticelli M.G., Forestieri R., Xia S., Joyasawal S., Lee T., Xu K., Smith I.A.B., Huryn D.M., Wilson R.B. (2020). Identification of a Novel Oleic Acid Analog with Protective Effects in Multiple Cellular Models of Friedreich Ataxia. ACS Chem. Neurosci..

[28] Batirel S., Yilmaz A.M., Sahin A., Perakakis N., Ozer N.K., Mantzoros C.S. (2018). Antitumor and antimetastatic effects of walnut oil in esophageal adenocarcinoma cells. Clin. Nutr..

[29] Negi A.S., Luqman S., Srivastava S., Krishna V., Gupta N., Darokar M.P. (2011). Antiproliferative and antioxidant activities of Juglans regiafruit extracts. Pharm. Biol..

[30] Johnston K., Sharp P., Clifford M., Morgan L. (2005). Dietary polyphenols decrease glucose uptake by human intestinal Caco-2 cells. FEBS Lett..

[31] Lyu S.-Y., Rhim J.-Y., Park W.-B. (2005). Antiherpetic activities of flavonoids against herpes simplex virus type 1 (HSV-1) and type 2 (HSV-2) in vitro. Arch. Pharmacal Res..

[32] Jakub T., Gazdová M., Smejkal K., Šudomová M., Kubatka P., Hassan S.T. (2020). Natural Products-Derived Chemicals: Breaking Barriers to Novel Anti-HSV Drug Development. Viruses.

[33] Pereira J.A., Oliveira I., Sousa A., Valentão P., Andrade P.B., Ferreira I.C.F.R., Ferreres F., Bento A., Seabra R.M., Estevinho L. (2007). Walnut (Juglans regia L.) leaves: Phenolic compounds, antibacterial activity and antioxidant potential of different cultivars. Food Chem. Toxicol..

[34] Al-Aboody M.S., Mickymaray S. (2020). Anti-Fungal Efficacy and Mechanisms of Flavonoids. Antibiotics.

[35] Nakatani N., Kayano S.-I., Kikuzaki H., Sumino K., Katagiri A.K., Mitani T. (2000). Identification, Quantitative Determination, and Antioxidative Activities of Chlorogenic Acid Isomers in Prune (Prunusdomestica L.). J. Agric. Food Chem..

[36] Parvez M.K., Alam P., Arbab A.H., Al-Dosari M.S., Alhowiriny T.A., Alqasoumi S.I. (2018). Analysis of antioxidative and antiviral biomarkers β-amyrin, β-sitosterol, lupeol, ursolic acid in Guiera senegalensis leaves extract by validated HPTLC methods. Saudi Pharm. J..

[37] Sa F.A.D.S., de Paula J.A.M., dos Santos P.A., Oliveira L.D.A.R., Oliveira G.D.A.R., Lião L.M., Paula J.R., Silva M. (2017). Phytochemical Analysis and Antimicrobial Activity of Myrcia tomentosa (Aubl.) DC. Leaves. Molecules.

[38] Müller U., Stübl F., Schwarzinger B., Sandner G., Iken M., Himmelsbach M., Schwarzinger C., Ollinger N., Stadlbauer V., Höglinger O. (2018). In Vitro and In Vivo Inhibition of Intestinal Glucose Transport by Guava (Psidium Guajava) Extracts. Mol. Nutr. Food Res..

[39] Vivek-Ananth R.P., Rana A., Rajan N., Biswal H.S., Samal A. (2020). In Silico Identification of Potential Natural Product Inhibitors of Human Proteases Key to SARS-CoV-2 Infection. Molecules.

[40] Luca S.V., Bujor A., Miron A., Aprotosoaie A.C., Skalicka-Woźniak K., Trifan A. (2020). Preparative separation and bioactivity of oligomeric proanthocyanidins. Phytochem. Rev..

[41] Han K.-I., Jung E.-G., Patnaik B.B., Hong C.-I., Kim Y.-J., Jung S., Han M.-D. (2017). Antibacterial and Antioxidant Activities of Leaf Extracts from Juglans sinensis, and its Phenolic Compositions. Nat. Prod. Commun..

[42] Hung P.-Y., Ho B.-C., Lee S.-Y., Chang S.-Y., Kao C.-L., Lee S.-S., Lee C.-N. (2015). Houttuynia cordata Targets the Beginning Stage of Herpes Simplex Virus Infection. PLoS ONE.

[43] Lobdell K.W., Stamou S., Sanchez J.A. (2012). Hospital-Acquired Infections. Surg. Clin. North. Am..

[44] Boev C., Kiss E. (2017). Hospital-Acquired Infections. Crit. Care Nurs. Clin. N. Am..

[45] Kaur K., Michael H., Arora S., Härkönen P.L., Kumar S. (2003). Studies on Correlation of Antimutagenic and Antiproliferative Activities of Juglans regia L. J. Environ. Pathol. Toxicol. Oncol..

[46] Jagtap A.G., Karkera S.G. (2000). Extract ofJuglandaceae regiaInhibits Growth, In-vitro Adherence, Acid Production and Aggregation ofStreptococcus mutans. J. Pharm. Pharmacol..

[47] Moghaddam P.Z., Mohammadi A., Feyzi P., Alesheikh P. (2017). In vitro antioxidant and antibacterial activity of various extracts from exocarps and endocarps of walnut. Pak. J. Pharm. Sci..

[48] Bisignano C., Mandalari G., Smeriglio A., Trombetta D., Pizzo M.M., Pennisi R., Sciortino M.T. (2017). Almond Skin Extracts Abrogate HSV-1 Replication by Blocking Virus Binding to the Cell. Viruses.

[49] Astani A., Reichling J., Schnitzler P. (2012). Melissa officinalis Extract Inhibits Attachment of Herpes Simplex Virus in vitro. Chemotherapy.

[50] Yang C.-M., Cheng H.-Y., Lin T.-C., Chiang L.-C., Lin C.-C. (2007). The in vitro activity of geraniin and 1,3,4,6-tetra-O-galloyl-β-d-glucose isolated from Phyllanthus urinaria against herpes simplex virus type 1 and type 2 infection. J. Ethnopharmacol..

[51] Lee S., Lee H.H., Shin Y.-S., Kang H., Cho H. (2017). The anti-HSV-1 effect of quercetin is dependent on the suppression of TLR-3 in Raw 264.7 cells. Arch. Pharmacal Res..

[52] Hopkins J., Yadavalli T., Agelidis A.M., Shukla D. (2018). Host Enzymes Heparanase and Cathepsin L Promote Herpes Simplex Virus 2 Release from Cells. J. Virol..

[53] Auer G.K., Weibel D.B. (2017). Bacterial Cell Mechanics. Biochemistry.

[54] Exner M., Bhattacharya S., Christiansen B., Gebel J., Goroncy-Bermes P., Hartemann P., Heeg P., Ilschner C., Kramer A., Larson E. (2017). Antibiotic resistance: What is so special about multidrug-resistant Gram-negative bacteria?. GMS Hyg. Infect. Control..

[55] Caporarello N., Olivieri M., Cristaldi M., Scalia M., Toscano M.A., Genovese C., Addamo A., Salmeri M., Lupo G., Anfuso C.D. (2017). Blood–Brain Barrier in a Haemophilus influenzae Type a In Vitro Infection: Role of Adenosine Receptors A2A and A2B. Mol. Neurobiol..

[56] Saraiva M., Castro R.H.A., Cordeiro R.P., Sobrinho T.J.S.P., Amorim E.L.C., Xavier H.S., Pisciottano M.N.C. (2011). In vitro evaluation of antioxidant, antimicrobial and toxicity properties of extracts of Schinopsis brasiliensis Engl. (Anacardiaceae). Afr. J. Pharm. Pharmacol..

[57] Rudramurthy S.M., Singh S. (2020). Candida Infections in Immunocompetent Hosts: Pathogenesis and Diagnosis. Curr. Fungal Infect. Rep..

[58] Genovese C., Corsello S., Nicolosi D., Aidala V., Falcidia E., Tempera G. (2016). Alterations of the vaginal microbiota in the third trimester of pregnancy and pPROM. Eur. Rev. Med. Pharmacol. Sci..

[59] Gintjee T.J., Donnelley M.A., Thompson G.R. (2020). Aspiring Antifungals: Review of Current Antifungal Pipeline Developments. J. Fungi.

[60] Genovese C., Cianci A., Corsello S., Ettore G., Mattana P., Tempera G. (2019). Combined systemic (fluconazole) and topical (metronidazole + clotrimazole) therapy for a new approach to the treatment and prophylaxis of recurrent candidiasis. Minerva Ginecol..

[61] Genovese C., Pulvirenti L., Cardullo N., Muccilli V., Tempera G., Nicolosi D., Tringali C. (2018). Bioinspired benzoxanthene lignans as a new class of antimycotic agents: Synthesis and Candida spp. growth inhibition. Nat. Prod. Res..

[62] Sytykiewicz H., Chrzanowski G., Czerniewicz P., Leszczyński B., Sprawka I., Krzyżanowski R., Matok H. (2015). Antifungal Activity of Juglans regia (L.) Leaf Extracts Against Candida albicans Isolates. Pol. J. Environ. Stud..

[63] Noumi E., Snoussi M., Hajlaoui H., Valentin E., Bakhrouf A. (2009). Antifungal properties of Salvadora persica and Juglans regia L. extracts against oral Candida strains. Eur. J. Clin. Microbiol. Infect. Dis..

[64] Raja V., Ahmad S., Irshad M., Wani W., Siddiqi W., Shreaz S. (2017). Anticandidal activity of ethanolic root extract of Juglans regia (L.): Effect on growth, cell morphology, and key virulence factors. J. Med. Mycol..

[65] Jafer F.N., A Naser L. (2020). The Biological Activity of Aqueous and Methanolic Extracts of Juglans regia on Yeasts and Pathologic Bacteria. Arch. Clin. Microbiol..

[66] An R.-B., Kim H.-C., Tian Y.-H., Kim Y.-C. (2005). Free Radical Scavenging and Hepatoprotective Constituents from the Leaves of Juglans sinensis. Arch. Pharmacal. Res..

[67] Thevissen K., Ghazi A., de Samblanx G.W., Brownlee C., Osborn R.W., Broekaert W.F. (1996). Fungal Membrane Responses Induced by Plant Defensins and Thionins. J. Biol. Chem..

[68] Tay L.Y., Jorge J.H., Herrera D.R., Campanha N.H., Gomes B.P.F.A., dos Santos F.A. (2014). Evaluation of different treatment methods against denture stomatitis: A randomized clinical study. Oral Surg. Oral Med. Oral Pathol. Oral Radiol..

[69] Taplitz R.A., Kennedy E.B., Bow E.J., Crews J., Gleason C., Hawley D.K., Langston A.A., Nastoupil L.J., Rajotte M., Rolston K.V. (2018). Antimicrobial Prophylaxis for Adult Patients with Cancer-Related Immunosuppression: ASCO and IDSA Clinical Practice Guideline Update. J. Clin. Oncol..

[70] Chen C.-Y., Milbury P.E., Lapsley K., Blumberg J.B. (2005). Flavonoids from Almond Skins Are Bioavailable and Act Synergistically with Vitamins C and E to Enhance Hamster and Human LDL Resistance to Oxidation. J. Nutr..

[71] Schreck K., Melzig M. (2018). Intestinal Saturated Long-Chain Fatty Acid, Glucose and Fructose Transporters and Their Inhibition by Natural Plant Extracts in Caco-2 Cells. Molecules.

[72] Marbaniang C., Kma L. (2018). Dysregulation of Glucose Metabolism by Oncogenes and Tumor Suppressors in Cancer Cells. Asian Pac. J. Cancer Prev..

[73] Reckzeh E.S., Karageorgis G., Schwalfenberg M., Ceballos J., Nowacki J., Stroet M.C., Binici A., Knauer L., Brand S., Choidas A. (2019). Inhibition of Glucose Transporters and Glutaminase Synergistically Impairs Tumor Cell Growth. Cell Chem. Biol..

[74] Taviano M.F., Miceli N., Acquaviva R., Malfa G.A., Ragusa S., Giordano D., Cásedas G., Les F., López V. (2020). Cytotoxic, Antioxidant, and Enzyme Inhibitory Properties of the Traditional Medicinal Plant Matthiola incana (L.) R. Br. Biol..

[75] Trandafir I., Cosmulescu S., Botu M., Nour V. (2016). Antioxidant activity, and phenolic and mineral contents of the walnut kernel (Juglans regia L.) as a function of the pellicle color. Fruits.

[76] Akbari V., Jamei R., Heidari R., Esfahlan A.J. (2012). Antiradical activity of different parts of Walnut (Juglans regia L.) fruit as a function of genotype. Food Chem..

[77] Cutrí C.C., Garozzo A., Siracusa M.A., Sarvá M.C., Tempera G., Geremia E., Pinizzotto M.R., Guerrera F. (1998). Synthesis and antiviral activity of a new series of 4-isothiazolecarbonitriles. Bioorganic Med. Chem..

[78] (2019). Performance Standards for Antimicrobial Susceptibility: M100—Performance Standards for Antimicrobial Susceptibility Testing.

[79] Clinical and Laboratory Standards Institute (2008). Reference Method for Broth Dilution Antifungal Susceptibility Testing of Yeasts, Approved Standard.

[80] Tenuta M.C., Deguin B., Loizzo M.R., Dugay A., Acquaviva R., Malfa G.A., Bonesi M., Bouzidi C., Tundis R. (2020). Contribution of Flavonoids and Iridoids to the Hypoglycaemic, Antioxidant, and Nitric Oxide (NO) Inhibitory Activities of Arbutus unedo L. Antioxidants.

[81] Malfa G.A., Tomasello B., Acquaviva R., la Mantia A., Pappalardo F., Ragusa M., Renis M., di Giacomo C. (2020). The Antioxidant Activities of Betula etnensis Rafin. Ethanolic Extract Exert Protective and Anti-diabetic Effects on Streptozotocin-Induced Diabetes in Rats. Antioxidants.

